# Cardiological Biopharmaceuticals in the Conception of Drug Targeting Delivery: Practical Results and Research Perspectives

**Published:** 2012

**Authors:** A.V. Maksimenko

**Affiliations:** Institute of Experimental Cardiology, Russian Cardiology Research and Production Complex, 3-rd Cherepkovskaya Str., 15а, Moscow, 121552 Russia

**Keywords:** drug targeting delivery, protein bioconjugates, thrombolytics, antithrombotic agents, molecular size of bioconjugates, density of molecular targets, enzyme connected antioxidants, cell adhesion molecules, pharmacological pre- and post-conditioning of myocardium

## Abstract

The results of the clinical use of thrombolytic and antithrombotic preparations developed on the basis of protein conjugates obtained within the framework of the conception of drug targeting delivery in the organism are considered. A decrease has been noted in the number of biomedical projects focused on these derivatives as a result of various factors: the significant depletion of financial and organizational funds, the saturation of the pharmaceutical market with preparations of this kind, and the appearance of original means for interventional procedures. Factors that actively facilitate the conspicuous potentiation of the efficacy of bioconjugates were revealed: the biomedical testing of protein domains and their selected combinations, the optimization of molecular sizes for the bioconjugates obtained, the density of target localization, the application of cell adhesion molecules as targets, and the application of connected enzyme activities. Enzyme antioxidants and the opportunity for further elaboration of the drug delivery conception via the elucidation and formation of therapeutic targets for effective drug reactions by means of pharmacological pre- and postconditioning of myocardium arouse significant interest.

## INTRODUCTION

Popular belief held that drugs can be delivered to a focus of pathological lesion via Paul Ehrlich’s ‘magic bullets’ [1]. This notion underpins the conception of drug-targeting delivery into the organism [2]; protein conjugates obtained via chemical and biological synthesis being among its objects [3, 4]. Thrombolysis became a significant area of the targeted extracellular application of these conjugates [5]. Successive decades have presented many opportunities for the results of the application of these agents (biopharmaceuticals) in thrombolytic and adjunctive therapy to be evaluated, as well as serving to outline the necessary directions for further biopharmacological innovations. This analytical review comprising data from PubMed, SCOPUS, Index Medicus/MEDLINE, and other databases, as well as the data of the Medical Research Library of the Russian Cardiology Research and Production Complex (Moscow), is devoted to the aforementioned issues.

## NEW DRUGS FOR THROMBOLYTIC THERAPY

The high prevalence of cardiovascular diseases is a well-known fact. In the Russian Federation, deaths due to these diseases account for more than half of the number of deaths [6]. Serious and overwhelming symptoms of cardiovascular disorders may appear either gradually or rather suddenly. The emergence of retrosternal pain (ischemic discomfort) is cause to suspect the progression of an acute coronary syndrome (ACS) [7]. Recording an electrocardiogram (ECG) enables one to reveal a mural or occlusive (completely blocking the vascular lumen) thrombus, on the basis of the ST-segment level in the ECG. The clinical diagnosis can be refined by determining the blood levels of creatine kinase (the MB isoform) and/or troponine (T or I) [7, 8]. Urgent thrombolytic therapy is required for patients with acute myocardial infarction.

Streptokinase (1.5 million IU for intravenous infusion for 30–60 min), alteplase (recombinant tissue plasminogen activator, 15 mg of the drug is given in the form of intravenous bolus (injection) followed by a 0.75 mg/kg infusion for 30 min and an additional 0.5 mg/kg infusion for 60 min; the total amount of the drug administered being less than 100 mg), tenecteplase (a mutant form (mutein) of a tissue plasminogen activator; 30–50 mg of the drug is administered intravenously depending on the patient’s body weight – 60 and over 90 kg), and purolase (prourokinase, 2 million IU of the drug is given intravenously followed by infusion of 4 million IU for 30–60 min) are used as thrombolytic agents in Russia. According to the standards of medical care in Russia, alteplase (trade name Actilyse), streptokinase and prourokinase (purolase) (i.e., the thrombolytics with a bolus-infusion scheme of administration) are prescribed to patients with acute myocardial infarction (by order of the Ministry of Healthcare and Social Development № 582 dated August 2, 2006). The use of such bolus agents as tenecteplase (trade name Metalyse) currently being promoted on the Russian pharmaceutical market has thus far been sporadic.

**Fig. 1:**
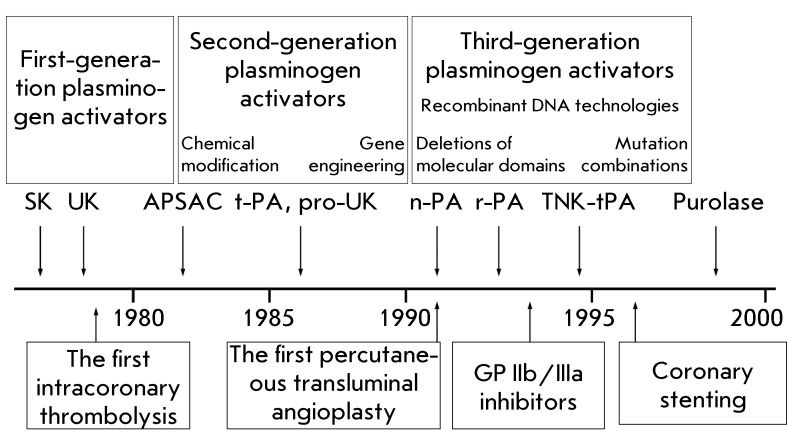
Chronology of the emergence of plasminogen activators of different generations and angioplasty means (balloons, guidewires, stents) of reperfusion therapy in clinical practice.

It should be noted that streptokinase (SK), the protein product of β-haemolytic streptococci, along with urokinase (UK), belongs to the first generation of plasminogen activators, whereas the tissue plasminogen activator (t-PA) and prourokinase (u-PA, pro-UK) belong to the second generation [9]. It is now possible to produce plasminogen activators in the form of nonglycosylated derivatives (Actilyse, purolase), owing to genetic engineering ( *Fig. 1* ). At present, tenecteplase (TNK-tPA, Metalyse) and reteplase (r-PA, Retavase) are third-generation clinically used plasminogen activators. The promotion of these pharmaceuticals toward clinical application emphasizes the specific features of modern biopharmacology and biotechnology, such as the significant amount of time required to design the pharmaceuticals and high cost of the resulting product (the price of an effective dose of the preparation is 2,000–3,000 USD). A number of new forms of plasminogen activators (anisoylated plasminogen/streptokinase activator complex – APSAC, lanoteplase – n-PA (mutant t-PA, mutein t-PA)) have not been widely used, because of a number of therapeutic indices; the alternatives (r-PA, TNK-tPA, purolase) are increasingly used.

Retavase (r-PA) is recommended for sequential double-bolus administration to patients with acute myocardial infarction. This pharmaceutical is a nonglycosylated t-PA with several domains (the finger-like domain, and a domain which is homologous to the epidermal growth factor, and the kringle domain 1) deleted from its molecule [10]. As a result of this modification, r-PA is capable of swift action, remaining in the bloodstream for a considerable time, and causing a lower depletion of the level of haemostatic blood proteins (systemic action) in comparison to the parent form of t-PA. Tenecteplase has a similar positive action (it is characterized by poorer suppression of the activity of plasminogen activator inhibitor type 1 and by a reduced contribution to fibrinogenolysis). The combination of mutations in the t-PA molecule (T103N, N117Q, KHRR(296–299)AAAA substitutions) was responsible for the emergence of the aforementioned properties and enabled the design of a pharmaceutical that is efficient after a single-bolus intravenous administration to patients with acute myocardial infarction [11, 12]. Targeting of the r-PA and TNK-tPA derivatives to thrombus (implementation of the targeting drug delivery concept) was successfully performed not via the use of an external vector (e.g., antifibrin monoclonal antibodies or their fragments) but by selection of the mutant forms of t-PA and the isolation of its domains. A normal t-PA molecule consists of several structural domains [9]: a fibronectin finger-like domain responsible for the high affinity binding to fibrin; the domain homologous to the epidermal growth factor which ensures the receptor binding to hepatic cells and accelerated clearance; and two kringle domains, one being essential for the binding of the domain 1 to endothelial cell receptors, and the second being responsible for low-affinity binding of domain 2 to fibrin. In addition, t-PA comprises the proteinase domain with plasminogen-specific activity. The proteinase domain contains the binding region of plasminogen activator inhibitor type 1. The molecular weight of this single-chain glycoprotein is ~ 64 kDa. Tenecteplase (Metalyse) and reteplase produced from it using genetic engineering techniques facilitate the further development of thrombolytic therapy ( *Fig. 1* ). Thus, emergency medical aid (EMA) teams staffed with medical or nursing personnel performed pre-admission bolus thrombolysis using tenecteplase according to the improved two-stage regimen (using ECG cardiotelemetry) [13]. The efficacy of the thrombolytic therapy was considerably determined by the symptom-to-needle time; its average value being 1 h 58 min. The door-to-needle time (from the time when the emergency medical aid team arrived to the injection) was 16 min. The noticeable reduction in time up to the beginning of therapy helped in the efficient treatment of 51.5% of the patients (one of the criteria was a decrease in the ST segment in ECG by more than 50% in the lead characterized by the greatest rise). The so-called interrupted myocardial infarction (when the ST segment decreases to the ECG isoline) was observed in 18.2% of the patients. In the presence of the EMA team, the lethality was 1.5%, and the lethality was 3.0% and 1.5% during the first 1 and 30th days, respectively. Thus, the lethality indicators did not increase and provided a significant decrease in the time taken to start the treatment due to the thrombolytic therapy performed by EMA teams, which can considerably improve the prognosis in patients with an acute myocardial infarction with a rise of the ST segment in ECG [13]. With allowance made for the necessity of settling the question regarding the price of tenecteplase pharmaceuticals, instrumentation of the EMA teams, organization of cardiac telemetry centres, and personnel training and education, this approach to providing the earliest thrombolytic treatment appears to be efficient and to undoubtedly help in the struggle against acute cardiovascular diseases.

## FORMATION AND DEVELOPMENT OF REPERFUSION THERAPY

The formation of thrombolytic therapy has revolutionized the treatment of acute myocardial infarction. It is noteworthy that as recently as the middle of the XXth century, the level of lethality amongst hospitalized patients was 30–40 %, a figure that has been reduced by almost 50% (14–17%) due to the increased use of intensive therapy wards [14]. The development of thrombolytic therapy has contributed to a considerable reduction in the mortality rate (to the level of 6–8%). The demand for further reduction of lethality levels due to myocardial infarction has fuelled the need for the establishment and improvement of reperfusion therapy, which is based on the use of thrombolytic drugs, methods and tools of transluminal balloon angioplasty, as well as coronary stenting ( *Fig. 1* ). The efficiency of intervention methods for bloodstream recovery via mechanical action have appeared to be rather high; however, some limitations still exist. According to the European Society of Cardiology Guidelines, emergency medical aid is to be urgently rendered to patients with acute cardiovascular diseases. “Five doors” are to be quickly passed through: the house doors (1), consultation/examination by a general physician (2), emergency medical aid manager (3), rendering emergency medical aid and transportation of a patient by the EMA team (4), and admission to a hospital/vascular centre (5) in order to receive qualified treatment. The beginning of therapy is delayed because of slow requests for medical aid and by heavy traffic, which can determine different time intervals (from symptom manifestation to the beginning of therapy) for the selection of the treatment strategy [15]. Thrombolytic therapy can be performed by an EMA team during the pre-hospital stage [13]. In the future, it will be possible to provide self-aid thrombolytic therapy even at home. However, in spite of the fact that the vast majority of organizational problems are being solved slowly and irrespective of the current situation in the financial and medical spheres in Russia, thrombolysis and angioplasty are complementary rather than alternative methods [16]. This approach is determined by the existence of hospitals equipped with tools for vascular angioplasty and stenting, as well as the proximity of the patient to them; the actions of the EMA teams; and timely thrombolytic therapy (in particular, when percutaneous coronary intervention is infeasible). The combination of thrombolysis and angioplasty is used in a number of cases. The latter approach seems to have a higher potential at the current level of development of the Russian healthcare system. In general, the problems relating to patient education, the improvement of the organization of cardiology aid (the “five doors” approach) and its means (the design of new stents and thrombolytics) remain pressing. However, the diversity of reperfusion therapy procedures and the high costs associated with this type of therapy have reduced the attractiveness of the sector to investors, a point attested to by the results of current biomedical research, which is focused on thrombolytic pharmaceuticals.

## CURRENT RESEARCH IN THE FIELD OF TARGETED THROMBOLYTICS

**Fig. 2:**
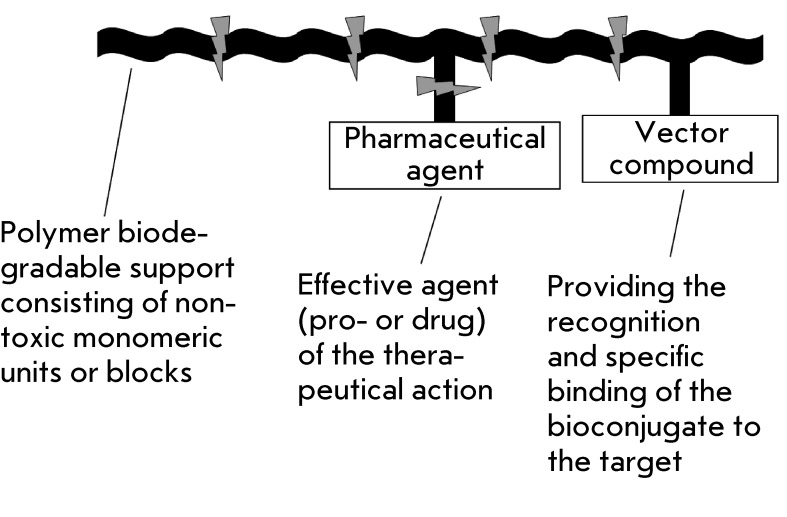
Schematic representation of the bioconjugate model for drug targeting delivery in the organism. The vector and drug components of the conjugate are covalently linked to the biodegradable matrix of a polymeric carrier.

The investigation of new thrombolytic agents [5, 9], whose intensive research began as recently as 15–20 years ago, has now considerably narrowed. The construction of targeted bioconjugates is based on the vector (which determines the recognition and binding to the target) and drug (ensuring the therapeutic effect) components bound to the biodegradable carrier matrix ( *Fig. 2* ). This model is currently being developed not as intensively as earlier. Antifibrin antibodies (or their fragments), fibrinogen (as a vector and carrier) or its components, as well as the complementary action of the combination of different t-PA and u-PA forms on the thrombus are no longer used. Vascular endothelial injury markers are now used as thrombotic leisure determinants [4]. Of course, their content in the blood and in other cell types that are available in the bloodstream should be low. Moreover, the density of their expression on endothelium should be sufficient for binding, which is required to achieve therapeutic effects and not result in negative side effects. Thus, the bioconjugate of urokinase with monoclonal antibodies (RE8F5) against the surface membrane protein of capillary pulmonary endothelium, which were bound via 4-succinimidyl-oxycarbonyl-α-methyl-α-(2-pyridyldithio)-toluene (SMPT) with retention of 85% of the initial urokinase activity was obtained for use in patients with pulmonary embolism [17]. With regards to the model for pulmonary embolism, this conjugate potentiated thrombolysis by 12–16 times, in comparison to urokinase and Retavase, without systemic activation of plasminogen and depletion of the fibrinogen level. Meanwhile, the covalent binding of the conjugate components via the disulphide bond (at its surface localization on the conjugate molecular structure) casts doubts on the conjugate’s stability and the potential for its practical development. The approach in the prevention of cerebrovascular thromboses appears to be of considerable interest [18]. The association of biotinylated t-PA with biotinylated erythrocytes via streptavidin induced rapid and long-lasting reperfusion in mice with cerebral thrombosis, as opposed to the effect of t-PA administered alone even at a dose tenfold higher [19]. The resulting adduct was characterized by an increased bloodstream half-life, and it was capable of lysing fresh thrombi (but not the old haemostatic plugs). In addition, the adduct exhibited a weaker response to the action of plasminogen activator inhibitor type 1 [20]. The erythrocytes proved to be efficient carriers of t-PA for thrombosis prevention; however, *ex vivo* modification was required for binding to t-PA, prior to introduction into the organism. This complicated modification can be avoided via the use of antibodies against erythrocyte membrane proteins. Thus, glycophorin A occurs on the erythrocyte surface. The use of the anti-glycophorin A single-chain antibody (scFv) within the recombinant protein form with low molecular weight single-chain urokinase, selectively activated by thrombine (scu-PA-T) [21] or with t-PA mutein (the kringle domain 2 and the protease domain) [22], ensures their binding to erythrocytes (40–95%) and considerably enhances the circulation time in the bloodstream (~35% of the dose administered remains in the bloodstream after 48 h). According to the results of these studies, the preventive delivery of various forms of plasminogen activators to erythrocytes can be considered as a new approach to the clinical prevention of thromboses, when the risk of vascular occlusion is high.

The low molecular weight recombinant single-chain urokinase plasminogen activator (lmw-scu-PA), fused with a single-chain variable antibody fragment (scFv) against a platelet endothelial cell adhesion molecule (PECAM-1), was obtained [23]. It was demonstrated, using the fused protein as an example, that cell adhesion molecules located on endothelium can act as targets for drug delivery. The recombinant form of prodrug lmw-scu-PA-scFv was bound specifically to the cells expressing PECAM-1 [23] and became a fibrinolytically active tcu-PA form after the cleavage of the Lys158–Ile159 bond in the urokinase fragment (lmw-scu-PA) by plasmin (at the sites of thrombus formation). Following the intravenous administration, the drug accumulated in the lungs of wild-type mice (but not those of the PECAM-1 knockout-mice) and was vastly more efficient than that exhibited by lmw-scu-PA. The drug was capable of lysing pulmonary emboli, as well as rapid removal from the bloodstream. These facts attest to the high potential of using fused proteins based on cell adhesion molecules and plasminogen pro activators to prevent thrombosis [4, 23].

A research group from the University of Pennsylvania (United States) led by V.R. Muzykantov [4, 19–23] is focusing on the sequential study of bioconjugates with a targeted fibrinolytic effect. Other research groups have either changed the direction of their studies or have released data sporadic ally[17, 18]. The questions relating to the immunogenicity of the recombinant forms, their applicability in acute lesions, and the development of adverse reactions remain open. The fact that tenecteplase (Metalyse) and reteplase (Retavase) have appeared as pharmaceuticals instills hope for successful developments.

## TARGETED ANTITHROMBOTIC DRUGS IN CLINICAL PRACTICE

**Fig. 3:**
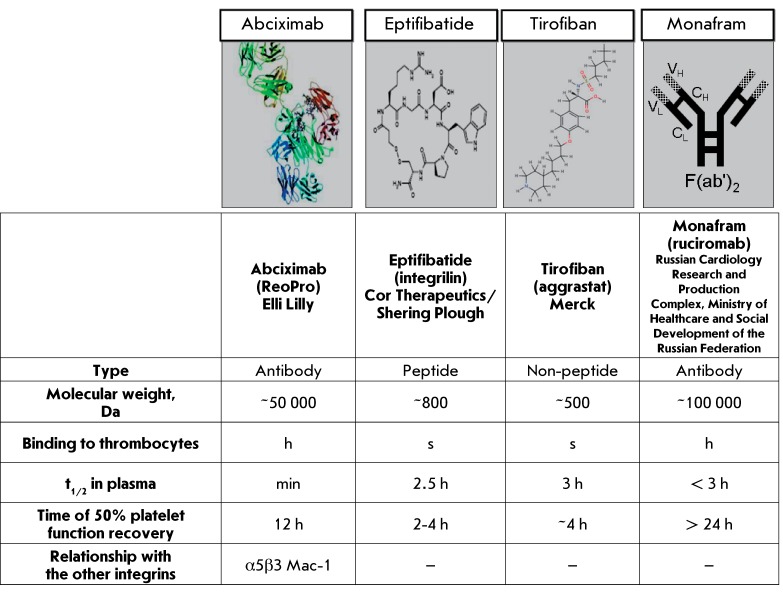
Molecular form and basic parameters of glycoprotein IIb/IIIa receptor blockers.

A large variety of antithrombotic drugs contribute to the stabilization of the effects of reperfusion therapy. These drugs include those with standard antithrombin effects (heparin, low molecular weight heparin (enoxaparin)), direct thrombin inhibitors (bivalirudin, dabigatran), factor Xa inhibitors – direct (apixaban, rivaroxaban, and otamixaban) and indirect ones (fondaparinux) [24], protease-activated receptor 1 (PAR-1) inhibitors, blockers inhibiting thromboxane A _2_ (TXA _2_ ) production (acetylsalicylic acid etc.), and P2Y _12_ receptor antagonists (clopidogrel, prasugrel, ticagrelor, cangrelor, etc.) [25]. The application of glycoprotein IIb/IIIa antagonists [26] for the inhibition of platelet aggregation during angioplasty in patients with acute coronary syndrome [27] is of interest from the viewpoint of the conception of drug targeting delivery (targeted to protein derivatives). Clinically available drugs are shown in *Fig. 3* . It should be noted that tirofiban and eptifibatid, which are currently moving towards certification on the pharmaceutical market, are considerably cheaper compared to abciximab and monafram (ruciromab being its non-patented name in Russia). The peptidomimetic tirofiban is a low-molecular-weight compound of non-peptide nature; eptifibatide is a small peptide. In contrast, abciximab consists of the Fab fragment of the recombinant chimeric antibody from the variable domains of the mouse anti-glycoprotein IIb/IIIa monoclonal antibody 7E3 and the constant domains of human immunoglobulin G; monafram is an F(ab’) _2_ fragment of anti-glycoprotein IIb/IIIa monoclonal antibodies. At the time of writing, competition for the extended use of the aforementioned drugs in clinical practice still exists. It should be noted that the antibody nature of abciximab and monafram enables the efficient recognition of these drugs by glycoproteins IIb/IIIa and binding to thrombocytes, which inhibits their aggregation.

Among efficient antithrombotic pharmaceuticals, fragments of protein molecules rather than the full-size molecules (identical to the case of third-generation plasminogen activators) are of interest for clinical practice [28]. In terms of a number of pharmacological properties, compounds with a molecular weight lower than 400 Da appear to be better compared to the larger types. Moreover, the lipophilicity of a compound under study is typically increased for the purpose of increasing the efficiency of a derivative and the specificity of its interaction with cell receptors, or ease of penetration through the membrane. However, this makes the compound less soluble. The compound becomes metabolically stable, serious adverse effects manifest themselves abruptly, and the level of toxicity increases (as follows from the results of the comparison of the toxicity of the compounds investigated in 1991 and 2000). The investigation of an enormous number of potential drugs has been discontinued for this reason [28]. 

Four levels of organization are conventionally recognized in the protein structure: the primary, secondary, tertiary, and quaternary structures. However, other gradations also exist [29]. According to them, the primary (amino acid sequence), secondary (α-helix, β-structure, etc.), supersecondary (ensembles of secondary structures interacting with each other: e.g., supercoiling of α-helices, i.e., coiling of two α-helices around one another) structures, structural domains (in particular, those determined by analyzing the electron density maps and corresponding to a 2.5-nm diameter globule, which satisfies the principle of easy coiling of a protein chain), globular proteins, and aggregates can be distinguished in a protein molecule. Nowadays, the priority in the design of biopharmaceuticals for cardiological purposes is on protein domains and their various combinations. However, this fact does not eliminate the necessity for a thorough investigation of their immunogenicity and toxicity.

## DEVELOPMENT OF DERIVATIVES FOR COMBINED ANTIOXIDANT THERAPY

The other approaches directed towards the retaining and enhancement of the effects of reperfusion therapy are to a larger extent associated with research studies as opposed to clinical ones. The antioxidants with trophicity to the lesion foci are being developed in order to block and reduce the adverse effect of oxidative stress, when excessive reactive oxygen species nonselectively damage molecules, tissues, and organs [30]. It is a newly forming area of antioxidant therapy, since the oxidative stress accompanies the development of cardiovascular disorders. Certain antioxidants (e.g., of vitamin or phenol nature) exhibit different clinical effects; meanwhile, oxidoreductases are notable for their high efficiency and specificity of their antioxidant action. Human superoxide dismutase (SOD), catalase (CAT), and glutathione peroxidase belong to the exhibiting antioxidant activity. SOD is represented by three isoforms: the cytosolic Cu,Zn-SOD (SOD-1), the mitochondrial Mn-SOD (SOD-2), and extracellular SOD (SOD-3, EC-SOD).

## EXTRACELLULAR SUPEROXIDE DISMUTASE

An increased content of one of the types of reactive oxygen species, superoxide radical (O _2_ ·¯), was observed in the arteries of spontaneously hypertensive rats. The transfer of the EC-SOD gene to these rats improved the functioning of their endothelium and reduced arterial pressure [31]. It is assumed that the interaction between O _2_ ·¯ and NO initially occurs in the extracellular space [32]. Among all the antioxidant enzymes, only EC-SOD localizes on the vascular luminal surface where it interacts with heparan sulphate proteoglycan via its heparan-binding domain [30, 32]. EC-SOD can presumably be located along the entire depth of the vascular wall and also between the endothelium and the vascular muscle [33]. The introduction of heparin (at therapeutic concentrations) results in the release of the EC-SOD previously bound to endothelial and other cells into the bloodstream [32, 34]. The antioxidant effect of EC-SOD mainly manifests itself on the vascular wall rather than in the bloodstream volume [30, 32]. It was revealed that diseases of the coronary vessels in humans are associated with a reduced level of heparin-released EC-SOD [35, 36]. A positive correlation between the level of heparin-released EC-SOD, the content of high-density lipoprotein cholesterol, and age was noted [36]. The protective effect of EC-SOD was attributed to the protection of the NO vascular dilator, which diffuses from the endothelium to the guanylate cyclase of smooth muscle cells [30, 32, 37], which was confirmed with data obtained from a model of volume-dependent (high-volume) hypertension in mice (1 kidney, 1 clip) [38]. Meanwhile, the impairment of endothelium-dependent dilation, increased arterial pressure, and vascular oxidative stress are observed in wild-type and EC-SOD knockout mice. Recombinant EC-SOD reduced arterial pressure and enhanced NO biocompatibility in the aorta of wild-type and EC-SOD knockout mice; however, it did not reduce the arterial pressure in endothelial NO synthase knockout mice and in wild-type mice that had received a NO synthase inhibitor. These results provided an illustrative demonstration of the fact that the targeted vascular effects of the recombinant EC-SOD are NO-mediated [38] and, along with the other data [39–41], point to the significant role of this biocatalyst upon hypertension. In addition to atherosclerosis [30, 32] and hypertension, oxidative stress and enzymatic antioxidants play an important role in the development of diabetes mellitus and heart failure [32]. The broad protective effect of enzymatic antioxidants emphasizes the topicality of using them to design new agents for combined therapy.

## MODIFICATION OF SUPEROXIDE DISMUTASE

**Fig. 4:**
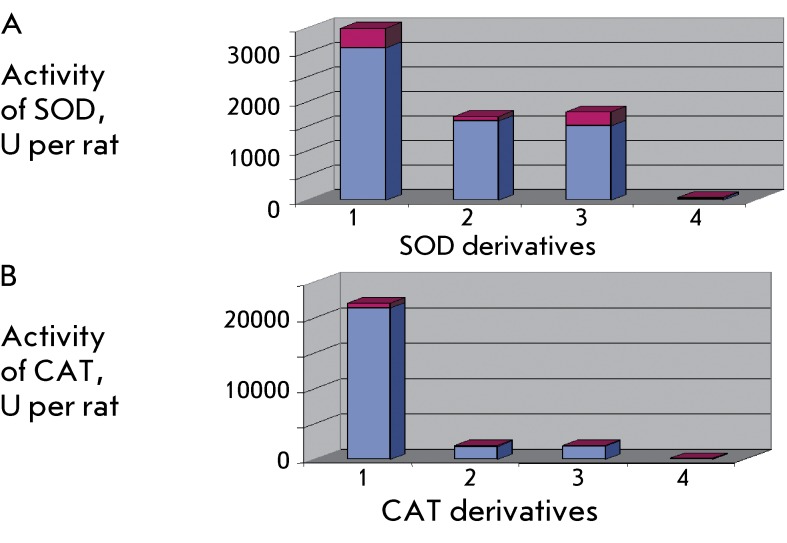
The comparison of the optimal dose intervals for the antithrombotic action of SOD (A) and CAT (B) derivatives. Designation: 1 – native enzyme, 2 – covalent conjugate of the enzyme with chondroitin sulphate, 3 – mixture of SOD-CHS and CAT-CHS derivatives, 4 – bienzyme SOD-CHS-CAT conjugate.

The low affinity of SOD-1 to membranes of the cells where reactive oxygen species are produced, its low stability in blood plasma, and the fact that it remained in the bloodstream for a short time is testament to the need for obtaining lecithinized SOD in which four phosphatidylcholine molecules would be covalently bound to the dimeric enzyme [42]. With the modification with lecithin, the SOD derivative exhibited an increased trophicity to the cell membrane; it reduced the lesion in mice with ulcerative colitis as early as 7 days after daily intravenous administration, whereas the native enzyme had to be introduced at 30-fold higher doses [42]. The considerably superior effect of using lecithinized SOD was also observed in mice with bleomycin-induced pulmonary fibrosis [43]. The targeting of protein agents to the lesion focus as a result of their modification is to a noticeable extent determined by the size of the resulting conjugates [44]. Thus, SOD conjugated with anti-PECAM-1 antibodies is characterized by optimal trophicity to lung endothelium when the conjugate is 300 nm in diameter. It is assumed that the targeting of the SOD conjugated with anti-PECAM-1 monoclonal antibodies to endothelial endosomes can have a pronounced anti-inflammatory effect [45].

## COMBINATION OF SUPEROXIDE DISMUTASE AND CATALASE ACTIVITIES

The inactivation of the endogenous enzyme by hydrogen peroxide [38] was revealed in the course of the investigation of the feasibility of using superoxide dismutases for antioxidant protection [32, 34, 46, 47]. The *in vivo * use of CAT (an intravenous bolus injection of the catalase–polyethylene glycol derivative for 3 days) reduced arterial pressure in wild-type spontaneously hypertensive mice (but not in the EC-SOD knockout ones) and improved the *ex vivo * function ofaortic endothelium. These data clearly attested to the key role of hydrogen peroxide in the inactivation of endogenous EC-SOD [38, 48]. The benefit of the reduction in the hydrogen peroxide level under oxidative stress conditions was demonstrated for the cell cultures. The super expression of CAT protected human aortic endothelium against apoptosis caused by the oxidized forms of low-density lipoproteins (oxLDL) [49]. These data attest to the fact that the simultaneous presence of SOD and CAT activity is reasonable to ensure protection against vascular oxidative stress. Different forms of these enzymes (both in the form of a mixture and in the form of conjugates) were used for this purpose.

## LINKAGE OF SUPEROXIDE DISMUTASE AND CATALASE

The results of the combined application of native forms of SOD and CAT were rather inconsistent [30, 46]. The simultaneous functioning of SOD and CAT in the focus of lesion development is required for the manifestation of a therapeutic effect [50]. This condition was fulfilled using a bienzyme conjugate in which SOD-1 was covalently bound to CAT via chondroitin sulphate (CHS) – a glycosaminoglycan of the vascular wall – to obtain the SOD-CHS-CAT adduct [46]. The conjugation changed the properties of SOD-1 by converting it into the SOD-3 form, which is the most similar to the glycoprotein [30, 51, 52]. In the resulting SOD-CHS-CAT conjugate, SOD and CAT catalyze two sequential reactions in which hydrogen peroxide (the SOD product) acts as a substrate for the reaction catalyzed by CAT and is converted into safe compounds: water and molecular oxygen (the reaction scheme is shown below):

O _2_ ·ˉ + O _2_ ·ˉ + 2H ^+^ → ^SОD^ H _2_ O _2_ + O _2_

H _2_ O _2_ + H _2_ O _2_ → ^CAT^ 2H _2_ O + O _2_

_____________________________________ 

4O _2_ ·ˉ + 4H ^+^ → ^COD/KAT ^ 2H _2_ O + 3O _2._

On the model of arterial thrombosis in rats induced via treatment of the vessel with a saturated solution of iron (II) chloride, the bienzyme conjugate SOD-CHS-CAT exhibited an antithrombotic effect when administered at doses lower by two orders of magnitude than those of the native SOD and CAT, and lower by an order of magnitudes than those of SOD and CAT (or their mixture) modified with chondroitin sulphate ( *Fig. 4* ) [50]. The linkage of proteins with CHS serves to target the bienzyme conjugate to the regions of the vascular lesion. It is known that the atherosclerotic lesion areas are characterized by an increased CHS content [30]. Early thickening of the intima of the vessel wall during atherogenesis is also associated with the accumulation of CHS [53]. After stents were mounted to New Zealand white rabbits with atherosclerosis, exposure of chondroitin sulphate proteoglycan was observed in the subendothelial arterial layer subjected to surgical intervention [54]. These data emphasize the feasibility and efficacy of using the components of vascular glycocalyx for drug-targeting delivery [53, 55].

## PHARMACOLOGICAL CONDITIONING OF myocardium 

**Fig. 5:**
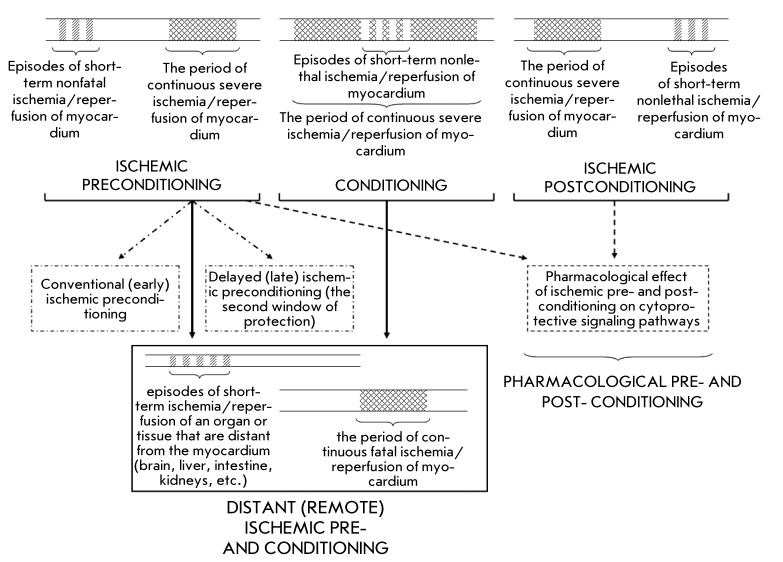
Schematic representation of different forms of ischemic pre- and post-conditioning of myocardium and its pharmacological conditioning.

**Fig. 6:**
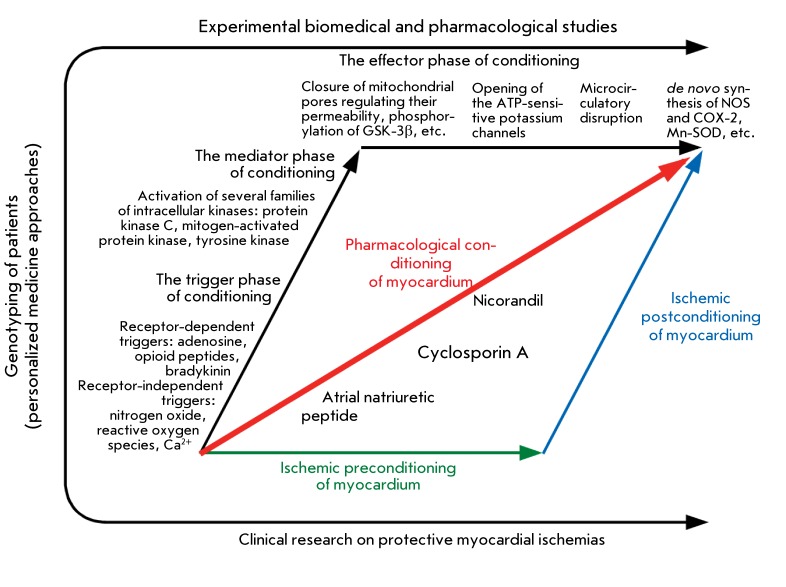
Translation of ischemic pre- and post- conditioning research related to biochemical and cell biology studies. Together, they constitute the resultant thrust of the pharmacological conditioning of myocardium in the frames of clinical and biomedical investigations integrated with increasing genotyping of patients.

The efficacy of pharmaceutical correction of the disorders of cardiovascular metabolism is also associated with another approach that is based on the production of a model for pharmacological interaction. As a result of using intermittent short-term episodes of ischemia/reperfusion before or after the period of severe, relatively continuous ischemia, the consequences of the disease turned out to be considerably less severe as compared to those without this procedure ( *Fig. 5* ). If the metabolic targets that are suitable for the successful pharmaceutical correction are determined after the mechanical actions upon myocardium (pre- and post-conditioning), it becomes possible to use the methods of pharmacological pre- and post-conditioning of myocardium ( *Fig. 6* ) [56]. Thus, in order to provide efficient interaction with a certain pharmaceutical, one needs to identify and prepare a target for cardivascular lesion that is sensitive to it.

## CONCLUSIONS

It should be noted that the interest in the research performed within the framework of the conception of targeted drug delivery and research focused on the development of bioconjugates for cardiology has waned. Such “truncated” protein forms as tenecteplase, reteplase, abciximab, and monafram have achieved clinical application. It is becoming apparent that it is necessary to change the vector component of bioconjugates when the antibodies against the markers of the lesion are being developed (cell adhesion molecules, glycocalyx components, etc.) rather than when thrombus components are applied at an increasing rate. The significance of the bioconjugate size, the density of the local accumulation of targeted markers in the focus of a developing lesion, and the use of a combined action of the catalysts of connected enzymatic reactions for efficient and specific drug targeting has now been revealed. The significance of the conception of targeted drug delivery, which is used to determine the strategy of bioconjugate construction, is decreasing. The modifications of the derivatives being designed acquire a significance; these modifications add useful properties (a lower effective dose, simplicity of use, and durable action) in addition to an appreciably high therapeutic effect and safety. New approaches to the conditioning of myocardium also emerge, facilitating the accurate identification and construction of significant targets for cardiovascular therapy. This will enable the modernisation of the conception of targeted drug transport during the development of cardiological biopharmaceuticals leading to the design of a new generation of targeting drugs.

The author expresses his sincere gratitude to Professors of the Russian Cardiology Research and Production Complex (Ministry of Healthcare and Social Development of the Russian Federation) E.P. Panchenko and I.I. Staroverov for their assistance with the selection and compilation of the data, as well as the discussion and presentation of this review.

The author is also deeply grateful to Academician E.I. Chazov and Corresponding member of the RAS V.N. Smirnov for his attention and support for the research and for the analytical efforts of his laboratory team. 

## Abbreviations

EC-SOD: extracellular superoxide dismutase
CAT: catalase
EMA: emergency medical aid
SOD: superoxide dismutase
CHS: chondroitin sulphate
SOD-CHS-CAT: covalent bienzyme superoxide dismutase chondroitin sulphat–catalase conjugate
ECG: electrocardiogram
